# *In Vivo* RNAi Rescue in *Drosophila melanogaster* with Genomic Transgenes from *Drosophila pseudoobscura*

**DOI:** 10.1371/journal.pone.0008928

**Published:** 2010-01-28

**Authors:** Christoph C. H. Langer, Radoslaw K. Ejsmont, Cornelia Schönbauer, Frank Schnorrer, Pavel Tomancak

**Affiliations:** 1 Max-Planck-Institute of Biochemistry, Martinsried, Germany; 2 Max-Planck-Institute of Molecular Cell Biology and Genetics, Dresden, Germany; VIB, Belgium

## Abstract

**Background:**

Systematic, large-scale RNA interference (RNAi) approaches are very valuable to systematically investigate biological processes in cell culture or in tissues of organisms such as *Drosophila*. A notorious pitfall of all RNAi technologies are potential false positives caused by unspecific knock-down of genes other than the intended target gene. The ultimate proof for RNAi specificity is a rescue by a construct immune to RNAi, typically originating from a related species.

**Methodology/Principal Findings:**

We show that primary sequence divergence in areas targeted by *Drosophila melanogaster* RNAi hairpins in five non-melanogaster species is sufficient to identify orthologs for 81% of the genes that are predicted to be RNAi refractory. We use clones from a genomic fosmid library of *Drosophila pseudoobscura* to demonstrate the rescue of RNAi phenotypes in *Drosophila melanogaster* muscles. Four out of five fosmid clones we tested harbour cross-species functionality for the gene assayed, and three out of the four rescue a RNAi phenotype in *Drosophila melanogaster*.

**Conclusions/Significance:**

The *Drosophila pseudoobscura* fosmid library is designed for seamless cross-species transgenesis and can be readily used to demonstrate specificity of RNAi phenotypes in a systematic manner.

## Introduction

Classical forward genetic mutagenesis screens pioneered the understanding of animal development in particular by using *Drosophila* as a model system [1]. The availability of the fly genome together with the discovery of RNA interference (RNAi) started an era of systematic reverse genetics, recently fuelled by the generation of genome-wide RNAi libraries in *Drosophila* [2], [3], [4]. Since RNAi can be achieved in a tissue specific manner in *Drosophila* [5] these genome-wide libraries have been used to study organ development [6], [7], [8]; and neuronal function [9] in an intact fly and will undoubtedly find many more applications in the near future.

A major pitfall of any RNAi approach are potential false positives resulting from unspecific knock-down of other genes than the anticipated target, the so called “off-target” effect. In case of randomly inserted hairpin transgenes false positives may arise from miss-expression of neighbouring genes. Despite the relatively low false positive rate in the systematic screens performed thus far (5–7%) ([7], [8]), its presence necessitates the confirmation of the association of a RNAi phenotype with a particular gene by an independent method. The best proof is the recapitulation of the RNAi phenotype by a classical mutant, however such an approach is not universal as mutants are either not available or may display un-interpretable, pleiotropic phenotypes. Alternatively, the RNAi phenotype can be confirmed by a second hairpin construct targeting a different region of the target gene that should show no or a different off-target effect. However, not all hairpins work to the same efficiency of knock-down and hence the observed phenotypes may differ despite the fact that only the correct on-target is knocked-down. Furthermore, not all genes are suited to generate several optimal 300 bp long hairpin sequences without overlap.

A conclusive proof of RNAi specificity is a rescue with a transgene that is immune to the RNAi and complements the loss of function of the target gene [10]. A convenient source of a RNAi-immune transgene is an orthologous gene from another closely related species that is divergent enough on the nucleotide sequence level to diminish RNAi efficiency while still functionally complementing the knock-down of the endogenous gene activity. This approach was successfully applied in human tissue culture RNAi using BAC transgenes from mouse [11] and in *C. elegans* with subcloned genomic BAC from *C. briggsae* [12]. When attempting RNAi rescue in living organisms, it is important to ensure that the rescue transgene gets expressed in the same cells and tissues in which RNAi was activated. Using the same driver for both RNAi and the gene rescue construct is one possibility, but the cDNA may not function properly when expressed from an artificial promoter. Recent advances in transgenesis of the *Drosophila* genome allow transformation of large BAC sized transgenes [13] and make it possible to test cross-species rescue using genomic transgenes that recapitulate endogenous gene expression patterns [14].

Here we evaluate computationally and experimentally the performance of genomic clones from non-melanogaster species in rescue of RNAi phenotypes in *Drosophila melanogaster (D. melanogaster)*. We identify *Drosophila pseudoobscura* (*D. pseudoobscura*) as a species suitable for RNAi rescue in terms of hairpin sequence divergence and make use of *D. pseudoobscura* FlyFos genomic fosmid library [15] to test RNAi specificity *in vivo*. We assayed for rescue of muscle specific knock-down phenotypes for five genes and were able to rescue three, suggesting that cross-species fosmid rescue is a useful strategy for establishing the specificity of RNAi phenotypes *in vivo* that can be easily applied to genome-wide RNAi screens in combination with the FlyFos library.

## Materials and Methods

### Bioinformatics Analysis of Hairpin Sequence Divergence

We downloaded pair-wise alignments between *D. melanogaster* and the 5 non-melanogaster species from the UCSC database (*D. melanogaster* release dm3 (UCSC)/Release 5 (FlyBase), non-melanogaster assembly releases by UCSC droSim1 (*D. simulans*), droAna3 (*D. ananassae*), dp4 (*D. pseudoobscura*), droPer1 (*D. persimilis*), droVir3 (*D. virilis*)). Using custom Perl scripts we extracted the portions of the pair-wise genome alignments covered by annotated Release 5 *D. melanogaster* transcripts (in case of multiple isoforms we selected the longest transcript to represent the gene) and collected the pair-wise alignment into single ‘multiple’ alignment file for each gene. These files were then searched with 12,591 hairpin sequences from genome wide transgenic RNAi library [2] (the library contains 15,059 hairpins; for simplicity only a single hairpin for each gene in the library was used for the analysis). 273 genes were not mapped because an alignment file was missing. Of the remaining genes 86% (10,858) mapped to the *D. melanogaster* sequence in the alignment files with 100% accuracy along the entire length of the hairpin. The 1733 hairpins that did not map completely were ignored in subsequent analysis. For the 10,858 fully mapped hairpins we counted the number of nucleotides conserved and the longest uninterrupted nucleotide stretch, both relative to *D. melanogaster* sequence. The collected counts were analyzed in Excel.

**Figure 1:**
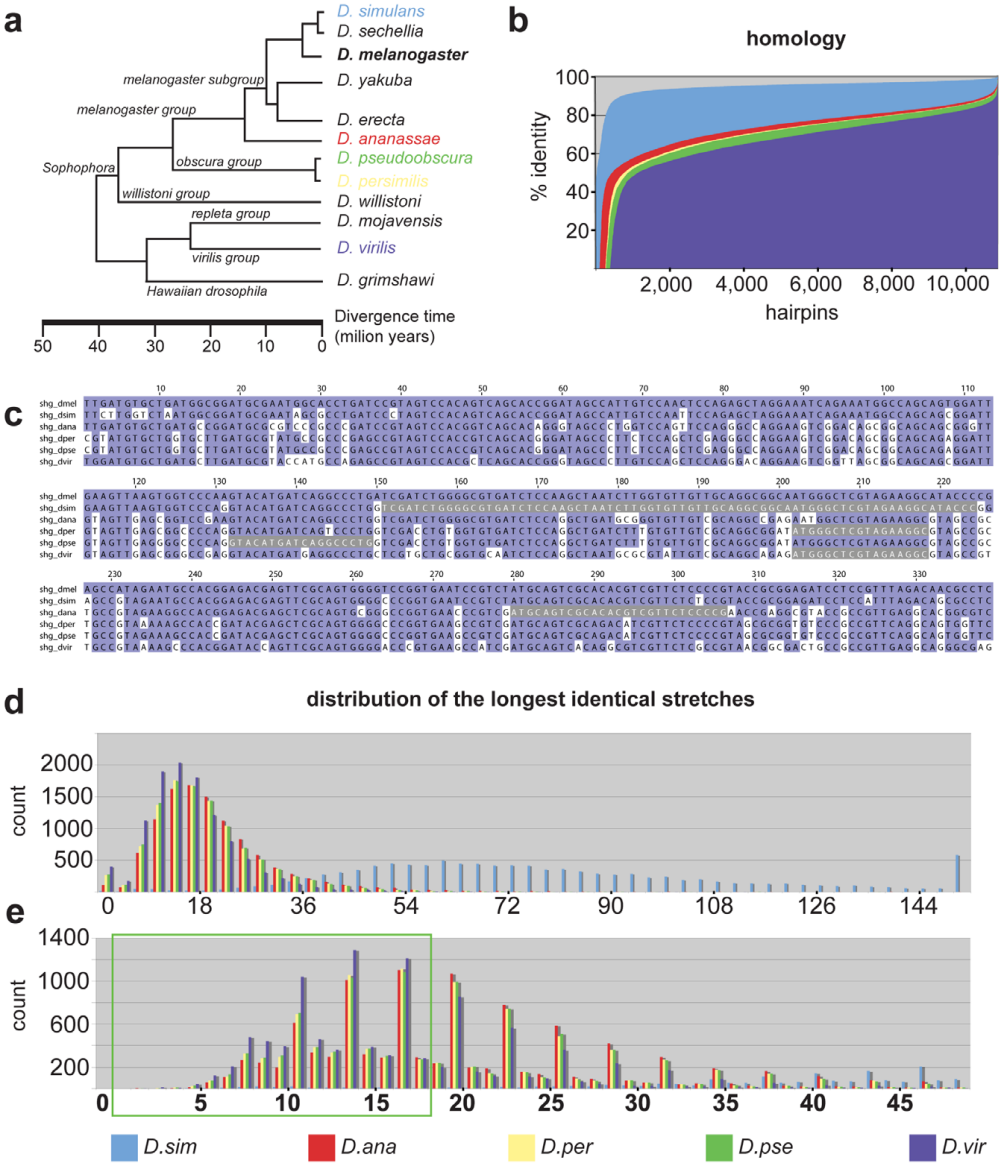
Comparative analysis of hairpin sequence divergence. (**a**) Phylogeny of sequenced *Drosophila* species. *D. melanogaster* is a standard model system in which transgenic RNAi is a well established technique. Species selected for comparative analysis are colour-coded. (**b**) Summary of the conservation of RNAi hairpins in pair-wise genome alignments with *D. melanogaster* as common reference. The percentage of nucleotides identical across the hairpin alignment (y-axis) is plotted for all hairpins ordered by increasing conservation (x-axis). Species are colour-coded according to (**a**). (**c**) An example of 6 species multiple sequence alignment for a hairpin targeting *shotgun (shg)*. Nucleotides identical to *D. melanogaster* are shaded in magenta. The longest uninterrupted stretch of identical nucleotides is shaded grey for each species. (**d**) Histogram of longest uninterrupted stretches for all hairpins binned in size groups of 3. (**e**) A portion of the histogram in (**d**) re-binned to bin size of 1 and limited to the maximum 50 nucleotide stretch. The periodic peaks are the consequence of the fact that most hairpins cover coding regions and reflect the increased likelihood of stretch interruption at the highly divergent third nucleotide of a codon triplet. The portion of the distribution that contains hairpins likely refractory to RNAi is highlighted by the green rectangle. The species are colour-coded as in (**a**).

**Figure 2:**
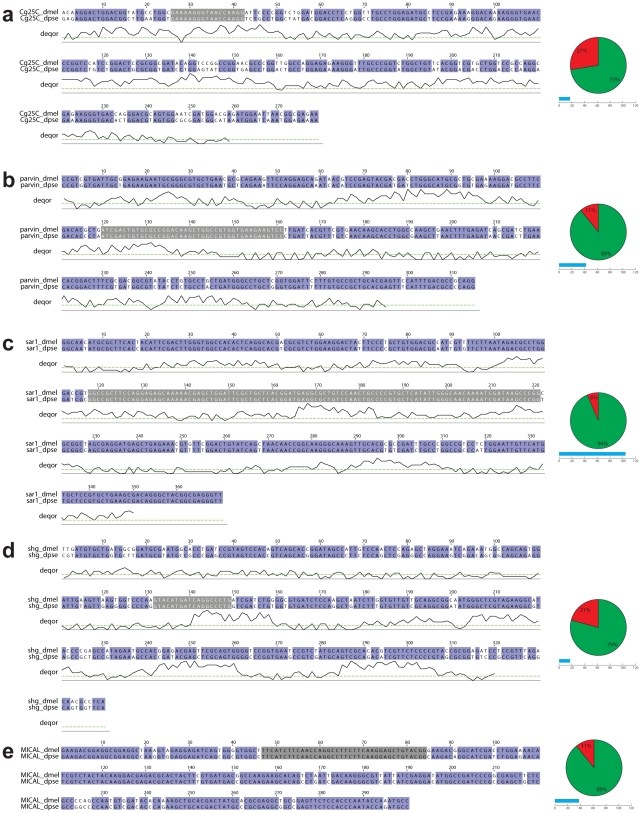
Pairwise sequence alignment of hairpins used in rescue experiments. Alignments between *D. melanogaster* and *D. pseudoobscura* for hairpins targeting (**a**) *Cg25c* (*collagen IV*), (**b**) *CG32528* (*parvin*), (**c**) *sar1*, (**d**) *shg* and (**e**) *Mical*. The extent of homology and the longest identical nucleotide stretch are graphically depicted next to each alignment. Matching nucleotides are shaded purple, mismatches white and the longest identical stretches are shaded grey within the alignments. The DEQOR scores are plotted below the alignments (**a–d**) and the score 5 cut-off above which the siRNA at that position is considered RNAi inefficient is depicted by a green line.

The multiple sequence alignments shown in **Figure 1c** and **Figure 2** were generated using EBI clustalw web-server and decorated in Jalview [16].

### Fosmid Selection and Transgenesis

At the time when the genes for the rescue experiments were selected we had mapped 2,592 *D. pseudoobscura* fosmids. These fosmids fully include 1278 predicted *D. pseudoobscura* genes with exactly one ortholog in *D. melanogaster* genome. The genome-wide transgenic RNAi screen for muscle phenotypes with *Mef2*-Gal4 driver resulted in 764 hits showing a defect in larval or flight muscle morphology [8]. 87 of these hits had a *D. pseudoobscura* ortholog covered by a fosmid and we manually selected five genes for the rescue experiment based on the RNAi phenotype and the placement of the ortholog within the fosmid (**Table 1** **and Figure S1**). Identifiers of the different data sources (fosmids, orthologs, RNAi hits) were matched using FlyMine [17]. The fosmid DNA was isolated as described in Ejsmont et. al. [15]. The transgenesis was performed by Genetic Services (http://www.geneticservices.com/).

**Table 1:** Overview of genes and fosmids.

*D. mel.* Gene	Trans-formant ID	FlyFos ID	RNAi phenotype	RNAi fosmid rescue?	Mutant allelic combination	Mutant phenotype	Mutant fosmid rescue?
*Cg25C (collagen IV)*	104536	045318	larval lethal	larval growth rescued; few pupa and adults	*Cg25C^k00405^/Df(2L)Exel7022*	embryo or larval lethal	n. a.
*CG32528 (parvin)*	11670	044975	myospheroid phenotype; early larval lethal	myospheroid phenotype rescued; 2x fosmid survive until early pupae	---	---	---
*sar1*	34191	045459	sarcomere defect; larval lethal	larval growth and sarcomere phenotype rescued; survive until early pupae	*sar1^05712^/Df(3R)ED6085*	embryo or larval lethal	few adult survivors (small size, can fly)
*shg*	27081	045685	missing flight muscles	no rescue	*shg^E17D^/shg^2^*	embryo or larval lethal	viable adults that fly
*Mical*	25372	045847	irregular flight muscle myofibrils	no rescue	*Mical^k1496^/Dr(3R)Exel6155*	Irregular flight muscle myofibrils	no rescue

Overview of all genes, RNAi constructs and fosmids used. The degree of homology between the genes in the targeted region is indicated. The RNAi and mutant phenotypes and their rescue by the fosmids is summarized.

### Fly Strains and Genetics

All crosses were done at 27°C to increase *GAL4* activity. All hairpins were obtained from the VDRC stock centre. All fosmids were inserted at the same site on the third chromosome (attP2 [18]) using site specific phiC31 integrase [19] and were recombined with *Mef2-GAL4* also located on the third chromosome [20]. Recombinants were easily identified by dsRed expression in the ocelli (expression in the eye is quenched by [white +]). If the hairpin was located on the third chromosome it was also recombined with the fosmid enabling to test for rescue in the presence of two copies of the fosmid. *Mical^k1496^* and *Mical^I666^* are described in [21], *Cg25C*, *sar1*, *shg* and *vkg* mutants as well as *Df(2L)Exel7022* deleting both *vkg* and *Cg25C* were obtained from Bloomington. A GFP trap in *CG6416* was used to label the Z-line in larvae [22]. *w[1119]* was used as wild type and is indicated by “+”. Recombinant chromosomes are indicated by “,”; homologous chromosomes by “/”.

### Phenotypic Analysis of Larval and Adult Flight Muscles, and Embryos

The larva-filets for immuno-stainings of larval muscles were prepared as described [23]. All dissections were done in relaxing solution (20 mM phosphate buffer, pH 7.0; 5 mM MgCl_2_, 5 mM EGTA, 5 mM ATP). Samples were fixed with 4% paraformaldehyde (PFA) in relaxing solution. Antibody incubations and subsequent washing steps were performed in PBS with 0,2% Triton X-100 instead of PBS-Tween. Samples were stained with rabbit anti-Kettin Ig 1/3 (1∶100) [24], mouse anti-Mhc 3e8 (1∶100) [25], mouse anti-Collagen IV (1∶100) [26], and rhodamine phalloidin or Alexa dye labelled secondary antibodies (Molecular Probes). To image flight muscles hemi-thoraces of adults were prepared by removing wings, head and abdomen with fine scissors, fixing the thoraces in 4% PFA in relaxing solution for 10 min and bisecting them sagitally with a sharp microtome blade. Thorax halves were then incubated in relaxing solution for 15 min, fixed for 10 min in PFA, washed twice in PBS+0,2% Triton X-100, incubated in rhodamine phalloidin (1∶500, in PBS +0,2% Triton X-100) for 30 min, washed two times in PBS +0,2% Triton X-100 and mounted in Vectashield. Embryos were fixed and stained as described [27] with rat anti-Mhc MAC147 (1∶100) (Babraham Institute) and mouse anti-CollagenIV (1∶100) [26]. Images were acquired with a Leica SP2 or Leica SP5 with 10x and 63x objectives to analyse flight muscles and myofibrils, and 40x objective to analyse embryos and larval muscles. Images were processed with ImageJ and Photoshop.

To analyse muscles of intact larvae the larvae carrying the *CG6416* GFP trap were immobilised by dipping in 65°C water for about 1 sec, and then mounted in 50% glycerol. Images were acquired on a Zeiss AxioImagerZ1 at 20x and analysed with ImageJ software.

To score for larval growth well fed, mated males and females were incubated in a vial for about 24 h, adults were removed and the vial was incubated for another 48 h or 72 h depending on the strength of the RNAi phenotype. All relevant crosses were done in parallel at the same time blind to the genotype. Larvae were immobilised by placing into 65°C water for about 1 sec, and then mounted in 50% glycerol. Images were acquired on a Leica M2FLIII with a ProgRes C14 at 1.25x magnification and larval length from head to tail was measured with Photoshop.

## Results

### Evaluation of Sequenced *Drosophila* Species for Transgenic RNAi Rescue Experiment

In order to identify the species best suited for RNAi rescue we performed comparative analysis of the divergence of *D. melanogaster* hairpin sequences in 5 different non-melanogaster species (**Figure 1a**) that sample the evolutionary tree of the sequenced *Drosophilid* genomes [28], [29]. We first mapped all hairpin sequences onto pair-wise, global genome alignments between *D. melanogaster* and the 5 non-melanogaster species available from UCSC [30] and extracted the percent identity for each pair (**Figure 1b**). As expected the pattern of hairpin sequence divergence follows the phylogeny; *D. simulans* sequences closely resemble *D. melanogaster* (94.75% are more than 90% conserved, i.e. 90^th^ percentile), the sister species *D. pseudooscura* and *D. persimilis* are almost indistinguishable (90^th^ percentile 1,78% and 1.63% respectively), *D. annanassae* similarity falls in between the *D. simulans* and the *obscura* group (90^th^ percentile 2,98%) and *D. virilis* is most divergent with respect to *D. melanogaster* (90^th^ percentile 0,41%). Overall the sequence homology of the species outside of *melanogaster* subgroup is quite comparable as 32.38% (*D.virilis*) to 55.61% (*D. annanassae*) of the hairpins have more then 75% of the nucleotides conserved relative to *D. melanogaster*.

We next asked how sensitive would the sequences from non-melanogaster species be to the *melanogaster* RNAi hairpins. It is broadly accepted in the RNAi field that stretches of 19 and more identical nucleotides can cause an ‘off-target’ effect [31], [32], [33]. Therefore we extracted the longest identity stretches from the pair-wise hairpin alignments for each species (**Figure 1c**) and analyzed their distribution. Vast majority (98.62%) of the longest identical stretches in *D. simulans* are longer then 18 nucleotides (**Figure 1d**) which allows us to conclude that this species would be a poor choice for *in vivo* RNAi rescue. Among the remaining species *D. virilis* has the largest proportion of hairpins that contain identity stretches shorter then 19 nucleotides (67.22%), making the clones likely refractory to RNAi. However the differences are not large; using the same criterion, 47.75% of *annanassae* clones, 53.58% of *D. persimilis* and 53.58% of *D. pseudoobscura* genes would also be refractory (**Figure 1e**). Altogether 81% of the genes in the VDRC hairpin collection have an ortholog with less then 19 nt identity stretch in at least one of the 5 *non-melanogaster* species. Since 94% of the refractory orthologs come from either *D. pseudoobscura* or *D. virilis* which are established model systems, we conclude that they are both well suited to serve as a donor for RNAi rescue experiment from the sequence divergence point of view.

Besides sequence divergence, the second important criterion for successful RNAi rescue is the ability of the transgene to complement the RNAi phenotype. The *D. virilis* life cycle is significantly longer then in *D. melanogaster* whereas *D. pseudoobscura* develops at a more similar pace [34]. Comparative micro-array time-course analysis of embryogenesis revealed that 24.7% of *D. virilis* genes exhibits differential gene expression profiles relative to *D. melanogaster* compared to 18.8% for *D. pseudoobscura* (P.T. manuscript in preparation). Based on these considerations we decided that *D. pseudoobscura* genomic transgenes are more likely to complement *D. melanogaster* loss-of-function phenotypes and are thus best suited for RNAi rescue.

### Selection of FlyFos Clones for *In Vivo* RNAi Rescue

We previously constructed a *D. pseudoobscura* genomic fosmid library, which we call FlyFos, in a vector containing 3xP3 dsRed dominant selection cassette [35] and attB sites for phiC31-mediated site-specific transgenesis [15], [18]. We thus far mapped end-sequences of 5,855 fosmid clones to *D. pseudoobscura* genome that cover 67.28% of the annotated *D. pseudoobscura* genes including at least 10 kb upstream and 5 kb downstream of the predicted gene model [15].

In order to select *D. pseudoobscura* FlyFos fosmids for RNAi rescue experiments we compared the complete list of hits from a genome-wide transgenic RNAi screen for muscle specific phenotypes induced by knocked-down with *Mef2-GAL4* driver [8], with the mapped *D. pseudoobscura* fosmids by linking annotated gene orthologs [36]. We selected five genes that lead either to larval lethality or a flightless phenotype (**Table 1**, see methods). All selected fosmids span at least to the next gene 5′ and 3′ from the gene assayed (**Figure S1**). The sequence similarity between *D. melanogaster* and *D. pseudoobscura* for the gene regions targeted by the used hairpins ranges from 73–94% (**Figure 2**). The largest stretch of exact match varies from 17–104 nucleotides. In order to estimate the ability of the siRNAs derived from the hairpins to function in RNAi we ran DEQOR analysis on the sequences [37] (**Figure 2**). DEQOR evaluates all possible 19mers from the hairpin sequence for a number of criteria (GC content, GC balance across the length of the siRNA and polynucleotide stretches) resulting in a score that reflects the efficiency of each 19mer in RNAi (the lower the score, the better RNAi performance, siRNAs below score 5 are considered suitable for RNAi). We used here DEQOR scores to ask whether the long identical stretches between *D. melanogaster* and *D. pseudoobscura* sequences are efficient in RNAi and thus likely to cross-silence the rescue transgene. Interestingly we found that most of the long identical stretch sequences (see **Figure 2c**) are predicted to perform poorly in RNAi suggesting that used hairpins will not significantly affect the *D. pseudoobscura* transgenes.

### *Drosophila pseudoobscura* Fosmids Rescue *In Vivo* RNAi Phenotypes in *Drosophila melanogaster*

We obtained *D. melanogaster* transgenics for all five fosmids by selecting for the dsRed expression in the eye, which is easily identifiable in *white^-^* genetic background. In case of the *Mical* fosmid instead of the eye we observed expression of dsRed in the thorax. As this fosmid was not able to rescue a *Mical* mutant allelic combination that recapitulates our observed RNAi phenotype, causing very irregular myofibrils in the indirect flight muscles (**Figure S2** and [21]), we judged this fosmid as non functional and did not investigate it further.

To test cross-species functionality of the *D. pseudoobscura* fosmid in *D. melanogaster* we rescued classical mutants of *shotgun* (*shg*) and *sar1* to viability and flight ability with the *shg* and *sar1* fosmids, respectively (**Table 1**) demonstrating that the *D. pseudoobscura* genes are fully functional in *D. melanogaster*.

For *shg* RNAi in muscle we observed a flightless phenotype caused by missing indirect flight muscles in the thorax [8]. The *shg* fosmid does not rescue this phenotype, indicating that the RNAi phenotype is either unspecific or the *D. pseudoobscura* gene is also targeted by the hairpin.

Three of our selected genes, the collagen IV homolog *Cg25C*, the parvin homolog *CG32528* and the small GTPase *sar1* lead to larval lethality upon knock-down with *Mef2-GAL4* ([8], **Table 1**). *Cg25C* is strongly expressed in embryonic hemocytes and supposedly has an important role in basement membrane function. We first analyzed P-element mutants to test if our collagen IV antibody recognizes Cg25C or Vkg, the second *Drosophila* collagen IV which is present in the basement membrane around the larval muscles [38]. As both genes face each other “head to head” the available P-elements located 5′ of each gene may also affect expression of the other more distant gene if enhancer elements are shared (**Figure S3a**). We find the expected strong collagen IV signal in hemocytes of wild-type stage 16 and stage 17 embryos with the collagen IV antibody (**Figure S3b,e**). This signal is absent in *Cg25C^k00405^/Df(2L)Exel7022* stage 16 and stage 17 embryos (**Figure S3c,f**). We also found no signal in *vkg^01209^/Df(2L)Exel7022* at stage 16, but detect a robust signal at stage 17 in these embryos (**Figure S3d,g**), suggesting that the collagen IV antibody does recognise Cg25C and not Vkg.

**Figure 3:**
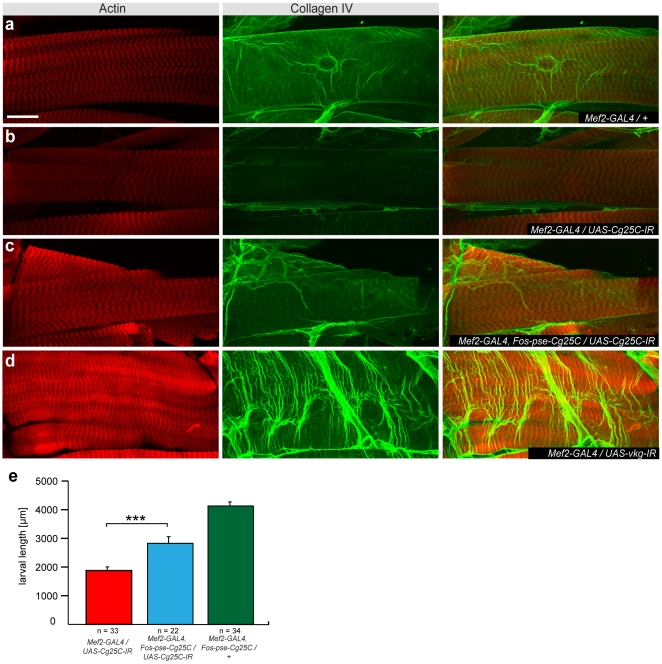
Rescue of *Cg25C* phenotype by *D. pseudoobscura* fosmid. (**a–d**) Collagen IV (green) wraps the larval muscles in wild type (**a**) and is strongly reduced in *Mef2-GAL4/UAS-Cg25C-IR* (TF104536) (**b**) but rescued by *FlyFos-pse-Cg25C* (**c**); Collagen IV levels are not altered in *Mef2-GAL4/UAS-vkg-IR* (TF106812) (**d**); actin is visualised with phalloidin; size bar corresponds to 25 µm. (**e**) Quantification of larval size in *Mef2-GAL4/UAS-Cg25C-IR* (TF104536) larvae (red) rescued by *FlyFos-pse-Cg25C* (blue) and wild type (green). ***p<0.0001 (unpaired two-tailed t-test). 72–96 h after egg laying were assayed. Error bars indicate standard error of the mean (SEM).

This conclusion is further corroborated by RNAi knock-down of both genes in muscle. We detect a collagen IV containing basement membrane around the growing larval muscles in wild type (**Figure 3a**). This collagen IV signal is severely reduced when *Cg25C* is knocked-down in muscle with *Mef2-GAL4* (**Figure 3b**) but not in *vkg* knock-down larvae (**Figure 3d**), which die at a comparable stage as *Cg25C* knock-down larvae [8]. This demonstrates that the collagen IV antibody indeed recognizes Cg25C and suggests an essential role for Cg25C in basement membrane function around growing muscles. The *D. pseudoobscura Cg25C* fosmid (*FlyFos-pse-Cg25C*) rescues larval growth significantly but not completely compared to knock-down and wild type (**Figure 3e**, **Table 1**) demonstrating the specificity of the RNAi knock-down. This incomplete rescue suggests that the *FlyFos-pse-Cg25C* fosmid is either not fully functional or not entirely immune to the *Cg25C* hairpin. Antibody staining against collagen IV/Cg25C argue for the latter as its localisation around the muscles is still markedly reduced in the rescued larvae (**Figure 3c**). In conclusion we demonstrate that the muscle specific RNAi knock-down of *Cg25C* can be rescued by the *FlyFos-pse-Cg25C*.

**Figure 4:**
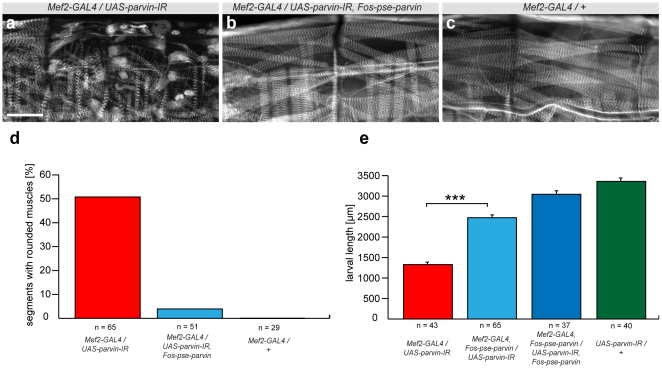
Phenotypic rescue of *parvin* by *D. pseudoobscura* fosmid. (**a–c**) Rounded/myospheroid muscle phenotype in *Mef2-GAL4/UAS-parvinIR* (TF11670) (**a**) is rescued by *FlyFos-pse-parvin* (**b**) to wild type (**c**); size bar corresponds to 100 µm. (**d**) Quantification of myospheroid phenotype rescue, percentage of segments containing rounded muscles are shown, below the total numbers of segments scored. (**e**) Quantification of larval size in *Mef2-GAL4/UAS-parvinIR* larva (red), rescued by one (light blue) or two copies of *FlyFos-pse-parvin* (dark blue), compared to wild type (green). Larvae 48–72 h after egg laying were assayed. Error bars indicate standard error of the mean (SEM), ***p<0.0001 (unpaired two-tailed t-test) compared to rescued larvae.

Muscles require the integrin complex for stable attachment to tendons [39]. We found that *parvin* knock-down results in early larval lethality with body muscles displaying a myospheroid phenotype (**Figure 4a**). This myospheroid phenotype is entirely rescued by the *D. pseudoobscura parvin* fosmid (*FlyFos-pse-parvin*) (**Figure 4b–d**). Similarly the growth defect in *parvin* knock-down larva is rescued; interestingly two copies of the fosmid increase the level of rescue (**Figure 4e** **and Figure S4**). We conclude that *Drosophila parvin* is required for muscle attachment, most likely via an integrin dependent mechanism as mouse parvin is an important member of the integrin complex [40] and integrin mutant *Drosophila* embryos display a myospheroid phenotype [39].

**Figure 5:**
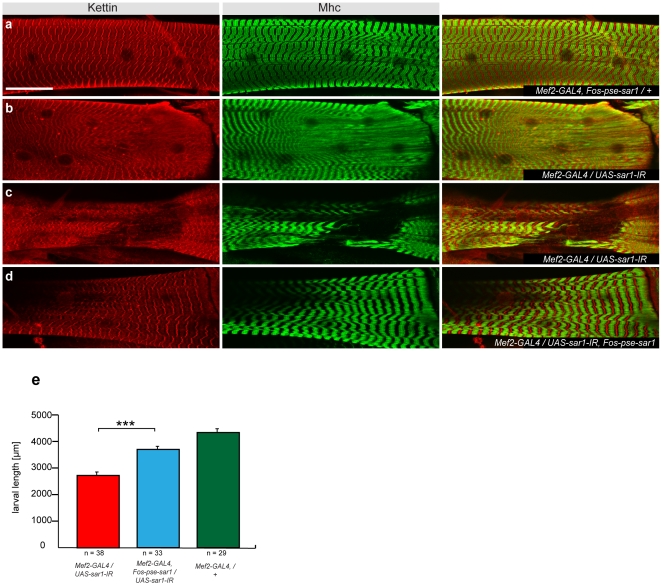
Phenotypic rescue of *sar1* by *D. pseudoobscura* fosmid. (**a–d**) Fading Z- and M-line or loss of sarcomeres in *Mef2-GAL4/UAS-sar1-IR* (TF34191) (**b, c**) is rescued by *FlyFos-pse-sar1*(**d**) to wild type (**a**). Z-lines are visualised with anti-Kettin (red), M-lines with anti-Mhc antibody (green); size bar corresponds to 50 µm. (**e**) Quantification of larval length in *Mef2-GAL4/UAS-sar1-IR* larvae (red), compared to *FlyFos-pse-sar1* rescued (blue) and wild type (green). Larvae 72–96 h after egg laying were assayed. Error bars indicate standard error of the mean (SEM), ***p<0.0001 (unpaired two-tailed t-test) compared to rescued larvae.

Finally we investigated the small GTPase *sar1* implicated in vesicle transport [41] and heart formation in the embryo [42]. Knock-down of *sar1* in muscle causes a muscle sarcomere phenotype. Both the myosin thick filaments and the Z-line anchoring the actin filaments show a “fading-Z” phenotype or in extreme cases we observe a partial loss of sarcomeres (**Figure 5a–c**). The *FlyFos-pse-sar1* completely rescues this sarcomere phenotype (**Figure 5d**) demonstrating a specific role of *sar1* for sarcomere formation and in turn larval growth (**Figure 5e**).

## Discussion

In this study we present a systematic evaluation of cross-species rescue with genomic transgenes for testing the specificity of transgenic RNAi knock-down in *Drosophila melanogaster*. We identified *D. pseudoobscura* and *D. virilis* as suitable, although not optimal, species for transgenic RNAi rescue and chose *D. pseudoobscura* FlyFos fosmid library to test the rescue performance. Despite the sequence similarity, which in some cases goes well beyond the 19 nt threshold (*sar1* 104 nt stretch), we were able to demonstrate rescue of the RNAi phenotype for three of the five genes tested. Similarly we showed rescue of classical mutants for *shg* and *sar1*. Overall, our strategy of cross-species RNAi rescue worked successfully for three of four cases in which the fosmid is functional.

We did not obtain a full rescue of the RNAi phenotypes. Since we observed full rescue of classical mutant phenotypes in two out of three cases and Kondo et. al [14] reported successful rescue in four out of four cases, we believe that in most cases the *D. pseudoobscura* gene products are able to functionally replace the *D. melanogaster* gene. We hypothesize that the incompleteness of the RNAi rescue is mainly caused by the sequence similarity of the genes between *D. melanogaster* and *D. pseudoobscura* which still results in knock-down of the *pseudoobscura* gene to some extent. In case of *parvin* we have strong evidence supporting this notion as two copies of the fosmid rescue better than a single copy. Kondo et. al. [14] reports full rescue of a rough-eye phenotype induced by over-expressing dsRNA directed against apoptotic gene *diap1* with an eye specific driver (GMR-GAL4) raising the possibility that the efficiency of the cross-species RNAi rescue will depend on the strength of the *GAL4* driver, the tissue and the gene tested.

Interestingly, the extent of the rescue does not necessarily correlate with the similarity of the hairpin-targeted sequences as measured by longest identity stretches (**Figure 2**, **and** **Table 1**). It appears that the ‘naïve’ application of 19 nt threshold generally thought to be sufficient for cross-silencing may strongly under-estimate the proportion of refractory orthologs. In contrary, data from cell culture indicate that even miss-matches every 12 bp can still result in some RNAi mediated silencing [43]. Hence assessing the efficiency of theoretical siRNAs generated from the hairpin by the DEQOR protocol may represent a more realistic measure of cross-silencing potential. Analysis of larger sets of cross-species rescue experiments will be required to evaluate the predictive power of the DEQOR analysis.

We observed a broad range of outcomes in our cross-species RNAi rescue experiments that allow us to define simple rules for their interpretation. We propose that if a phenotypic rescue, albeit incomplete, is observed, the specificity of the RNAi knock-down need not be questioned any longer. If, however, no rescue is observed, it is necessary to determine whether the rescuing construct is active. This can be done by rescuing a classical mutant allele if available, or by showing, using antibody staining or RNA *in situ*, that the expression of the hetero-specific transgene mimics the expression of wild-type ortholog and is unperturbed in the RNAi genetic background. For the purpose of visualizing the rescue construct in a straightforward manner, it may be useful to tag the construct with a reporter such as GFP [15]. When these controls establish that the rescue construct is functional, the absence of RNAi rescue indicates that the observed phenotype is caused by an off-target knock-down.

In the future we plan to establish a fosmid library for *D. virilis* to expand the spectrum of genes in which cross-species RNAi rescue is an option. However our bioinformatics analysis indicates that for approximately 1/3 of the genes even the distantly related *Drosophilids* diverged insufficiently to attempt cross-species RNAi rescue with confidence. It may be possible to optimize the placement of the targeting hairpin within the gene model to enable efficient cross-species rescue, but the existing transgenic RNAi libraries cannot benefit from this approach. Alternatively one can use recombineering manipulation to render *D. melanogaster* fosmid sequences RNAi immune by introducing silent mutations in the stretch covered by the hairpin [43]. Such strategy is costly and laborious despite the advances in high-throughput manipulation of large clones in bacteria.

The *D. pseudoobscura* fosmid library is freely available at http://transgeneome.mpi-cbg.de/. The rescue with FlyFos clones is very simple; once a suitable clone containing the gene of interest is identified, it can be directly injected into *D. melanogaster* without additional modification. Hence, our system is simpler than the fosmid retrofitting approach developed by Kondo et. al. [14]. After transgenesis, that can be efficiently performed by a company, the fosmids marked with dsRed in eyes and ocelli can be easily recombined with most existing GAL4 lines or hairpin constructs.

In conclusion, cross-species rescue is a valid approach to demonstrate RNAi specificity and thus may complement the vast number of *in vivo* RNAi studies done in *Drosophila* [6], [7], [8], [9]. It may go beyond the mere rescue of an RNAi loss of function phenotype as it can also be applied to perform structure-function analysis in an RNAi knock-down background [44]. The fosmids can easily be engineered by liquid culture recombineering to delete or modify specific protein domains or single critical amino acids [13], [15], [45]. This will enable systematic structure-function studies for genes, for which no mutants are available, or more importantly mutants that display highly pleiotropic phenotypes.

## Supporting Information

Figure S1 Genomic region of *D. pseudoobscura* fosmids. Screenshots of gbrowse representations of the genomic regions of *D. pseudoobscura* genome corresponding to extent of the fosmids used in rescue experiments. The gene orthologous to the *D. melanogaster* gene knocked-down by RNAi is marked by the presence of its transcript and CDS. The FlyFos identifier and mapping coordinates of end-sequences of the fosmid on *D. pseudoobscura* genome are shown on top of each gbrowse view.
(0.93 MB TIF)

Figure S2 Mical mutant and RNAi phenotype Indirect flight muscles (a–d) and myofibrils of these IFMs (e–g) in wild type (a, e) *Mical* mutants (b, f), *Mef2-GAL4/UAS-Mical-IR* (TF25372) (c, g) and *Mical* mutants carrying the *FlyFos-pse-Mical* (d, h). Actin is visualised by phalloidin; size bar in (a–d) corresponds to 100 µm, in (e–g) to 10 µm.
(2.19 MB TIF)

Figure S3 *Cg25C* and *vkg* genomic locus and collagen IV protein expression. (a) Screenshot of gbrowse representation of the genomic regions of *D. melanogaster Cg25C* and vkg; the position of the P-elements *vkg^01209^* and *Cg25C^k00405^* are indicated according to Flybase. (b–g) Stage 16 (b–d) and stage 17 (e–g) wild-type (b, e), *Cg25C^k00405^/Df(2L)Exel7022* (c, f) and *vkg^01209^/Df(2L)Exel7022* (d, g) embryos are stained for Mhc in green and Collagen IV in red; size bar corresponds to 50 µm.
(3.37 MB TIF)

Figure S4 Rescue of *parvin* knock-down. Larva of 48–72 h (a–c) or 72–96 h (d–f) were imaged at the same magnification. *Mef2-GAL4/UAS-parvinIR* (TF11670) (a, d) stay tiny compared to *Mef2-GAL4/UAS-parvinIR*, *FlyFos-pse-parvin* (b, e) and *UAS-parvin-IR/ +* control larvae (c, f). Size bar corresponds to 1 mm.
(1.94 MB TIF)
