# Supplementary figures and images for: In Vivo RNAi Rescue in Drosophila melanogaster with Genomic Transgenes from Drosophila pseudoobscura
Source: PLoS One. 2010 Jan 28;5(1):e8928. doi: 10.1371/journal.pone.0008928 (PMC2812509; doi:10.1371/journal.pone.0008928)

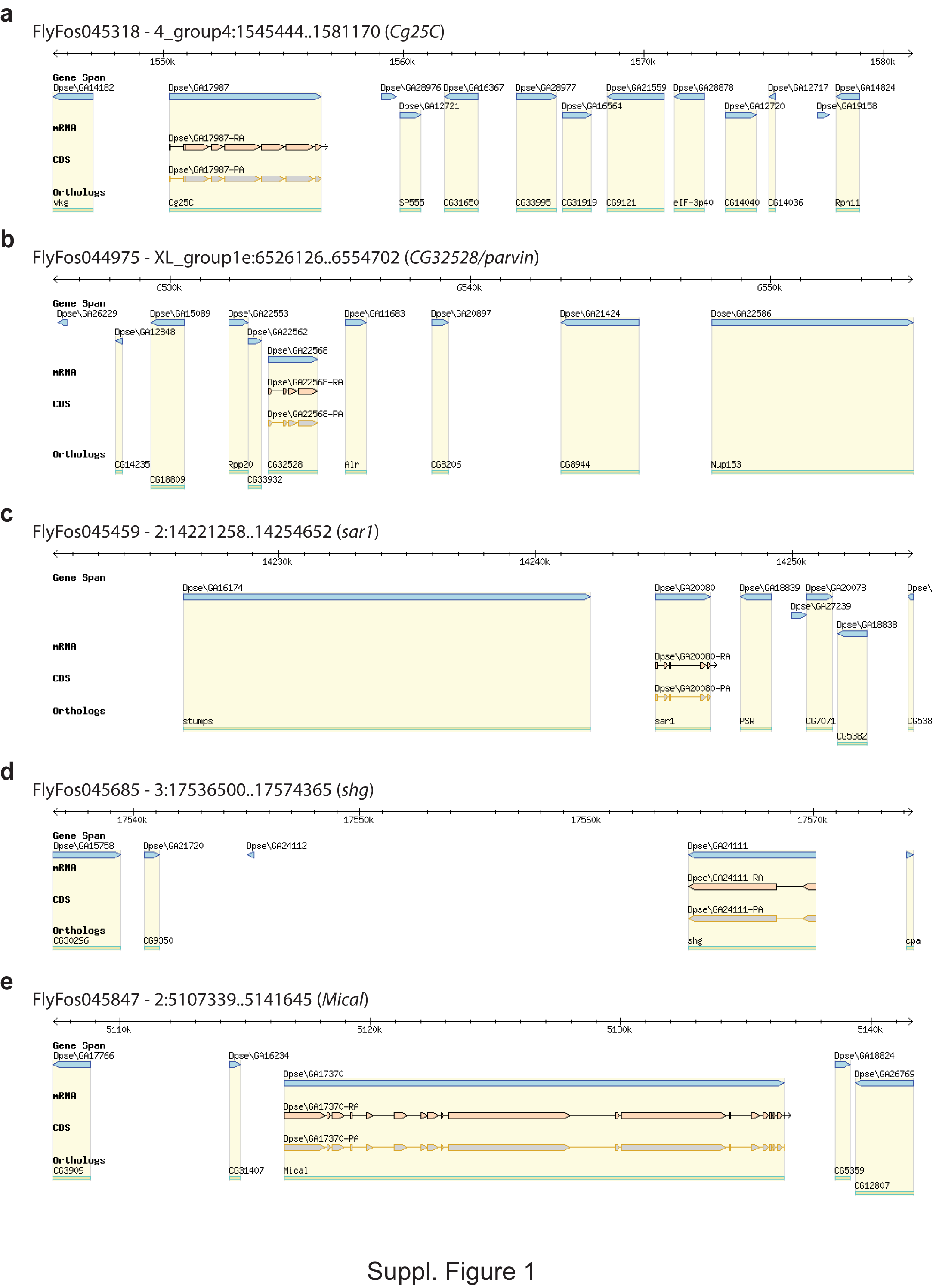

Supplement: Figure S1 — Genomic region of D. pseudoobscura fosmids. Screenshots of gbrowse representations of the genomic regions of D. pseudoobscura genome corresponding to extent of the fosmids used in rescue experiments. The gene orthologous to the D. melanogaster gene knocked-down by RNAi is marked by the presence of its transcript and CDS. The FlyFos identifier and mapping coordinates of end-sequences of the fosmid on D. pseudoobscura genome are shown on top of each gbrowse view. (0.93 MB TIF) [file pone.0008928.s001.tif]

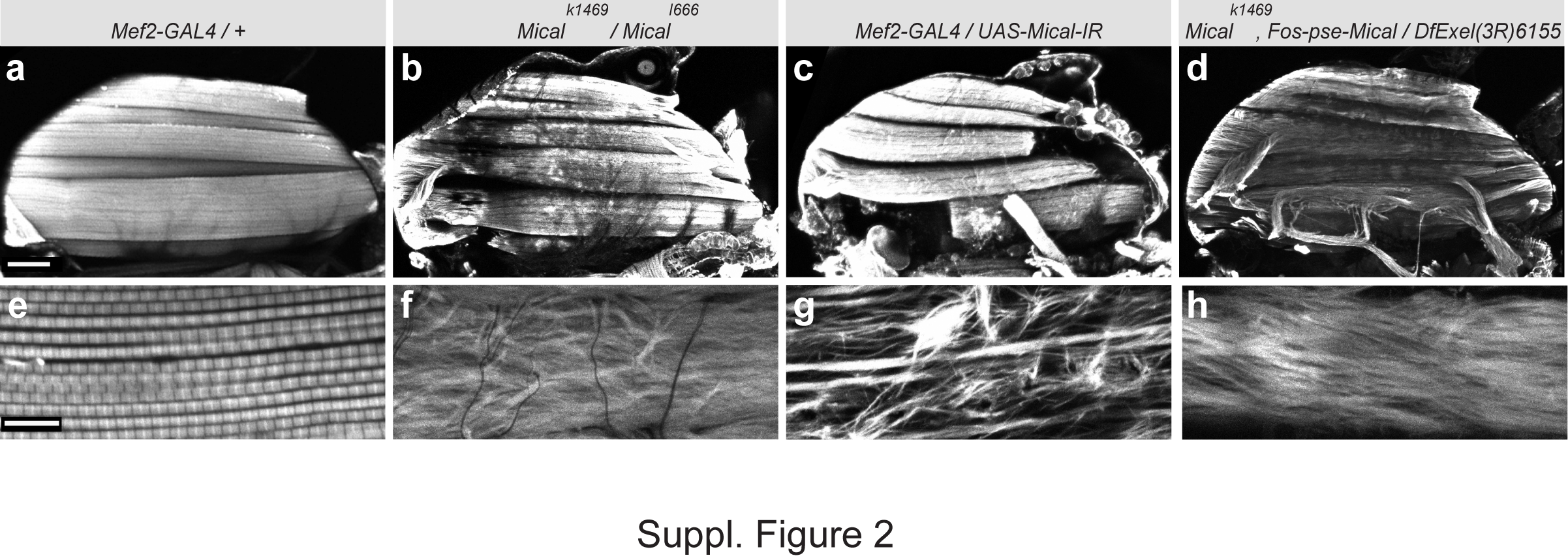

Supplement: Figure S2 — Mical mutant and RNAi phenotype Indirect flight muscles (a–d) and myofibrils of these IFMs (e–g) in wild type (a, e) Mical mutants (b, f), Mef2-GAL4/UAS-Mical-IR (TF25372) (c, g) and Mical mutants carrying the FlyFos-pse-Mical (d, h). Actin is visualised by phalloidin; size bar in (a–d) corresponds to 100 µm, in (e–g) to 10 µm. (2.19 MB TIF) [file pone.0008928.s002.tif]

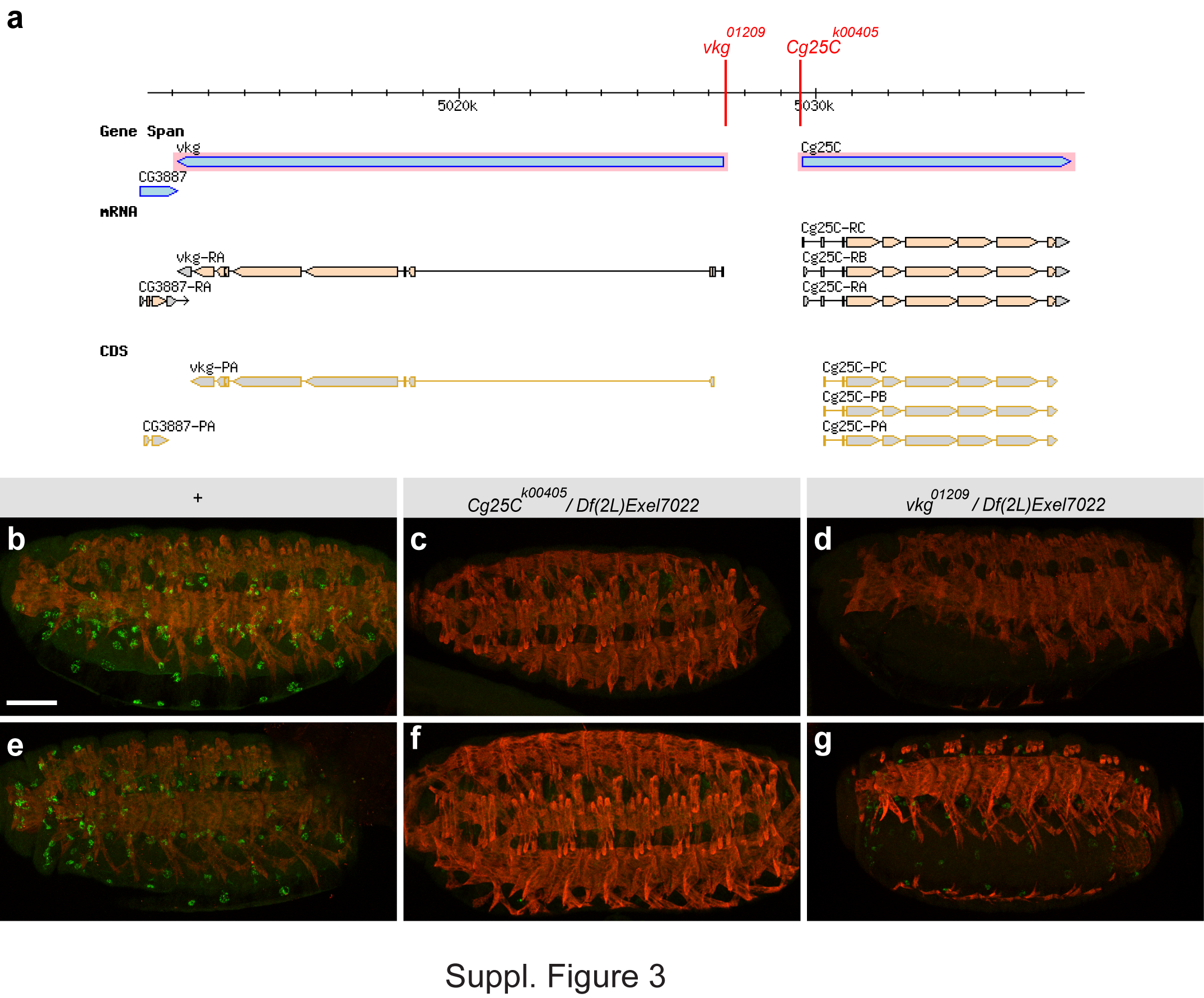

Supplement: Figure S3 — Cg25C and vkg genomic locus and collagen IV protein expression. (a) Screenshot of gbrowse representation of the genomic regions of D. melanogaster Cg25C and vkg; the position of the P-elements vkg01209 and Cg25Ck00405 are indicated according to Flybase. (b–g) Stage 16 (b–d) and stage 17 (e–g) wild-type (b, e), Cg25Ck00405/Df(2L)Exel7022 (c, f) and vkg01209/Df(2L)Exel7022 (d, g) embryos are stained for Mhc in green and Collagen IV in red; size bar corresponds to 50 µm. (3.37 MB TIF) [file pone.0008928.s003.tif]

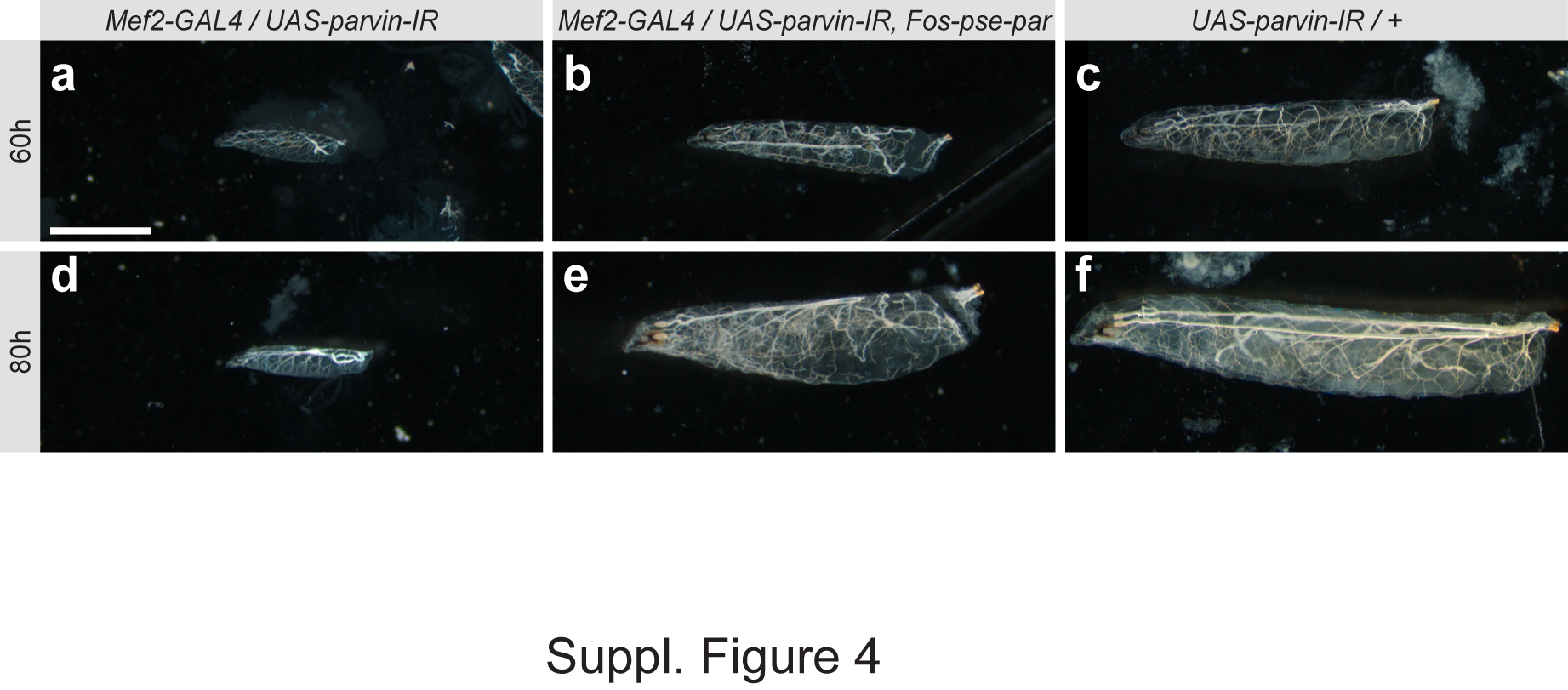

Supplement: Figure S4 — Rescue of parvin knock-down. Larva of 48–72 h (a–c) or 72–96 h (d–f) were imaged at the same magnification. Mef2-GAL4/UAS-parvinIR (TF11670) (a, d) stay tiny compared to Mef2-GAL4/UAS-parvinIR, FlyFos-pse-parvin (b, e) and UAS-parvin-IR/ + control larvae (c, f). Size bar corresponds to 1 mm. (1.94 MB TIF) [file pone.0008928.s004.tif]
